# An Association Between GLP-1 Receptor Expression on Regulatory T Cells and the Severity of Coronary Artery Stenosis and Inflammatory Dysregulation in Coronary Heart Disease

**DOI:** 10.31083/RCM39927

**Published:** 2025-09-25

**Authors:** Mingzhu Lv, Mengmeng Yue, Min Yang, Xiaolei Li, Kun Wu, Ting Yao, Hang Qian, Long Chen, Wenwen Wu, Xinwen Min, Handong Yang, Hao Xu, Aihua Mei, Jun Chen

**Affiliations:** ^1^Sinopharm Dongfeng General Hospital (Hubei Clinical Research Center of Hypertension), Hubei Key Laboratory of Wudang Local Chinese Medicine Research, Hubei University of Medicine, 442000 Shiyan, Hubei, China; ^2^Geriatrics Department, Sinopharm Dongfeng Huaguo Hospital, 442000 Shiyan, Hubei, China; ^3^The Second Clinical School, Hubei University of Medicine, 442000 Shiyan, Hubei, China; ^4^School of Public Health, Hubei University of Medicine, 442000 Shiyan, Hubei, China

**Keywords:** coronary heart disease (CHD), glucagon-like peptide-1 receptor (GLP-1R), regulatory T cells (Tregs), coronary artery stenosis severity, immunomodulation

## Abstract

**Background:**

Regulatory T cells (Tregs) play pivotal roles in immune homeostasis; however, the association between Tregs and the pathogenesis of coronary heart disease (CHD) remains unclear. Thus, this study aimed to investigate the relationships among Tregs, glucagon-like peptide-1 receptor (GLP-1R) expression, and CHD risk, with a focus on the severity of coronary artery stenosis and inflammatory cytokine dynamics.

**Methods:**

A total of 130 CHD patients (stratified by the Gensini score into low-/high-risk stenosis subgroups) and 70 non-CHD controls were enrolled in this case–control study. Peripheral blood Tregs (CD4^+^CD25^+^FoxP3^+^) and GLP-1R^+^ Tregs were quantified via flow cytometry. Plasma cytokines interleukin-2 (IL-2), interleukin-4 (IL-4), interleukin-6 (IL-6), interleukin-10 (IL-10), interleukin-35 (IL-35), and tumor necrosis factor-alpha (TNF-α) were measured. Multivariate logistic regression and receiver operating characteristic (ROC) analyses were employed to evaluate the associations between Treg subsets and clinical outcomes; meanwhile, the Spearman correlation was used to assess the relationships between cytokines.

**Results:**

CHD patients presented with significantly lower proportions of total Tregs (*p* < 0.001) and GLP-1R+ Tregs (*p* = 0.013) compared to controls, with further reductions in the high-risk stenosis subgroups. Multivariate analysis identified both Tregs (CHD: odds ratio (OR) = 0.752; *p* < 0.001; stenosis: OR = 0.760; *p* = 0.021) and GLP-1R^+^ Tregs (CHD: OR = 0.859; *p* = 0.013; stenosis: OR = 0.840; *p* = 0.040) as independent predictors. The ROC analysis demonstrated diagnostic utility for Tregs (CHD: area under the curve (AUC) = 0.663; stenosis: AUC = 0.635) and GLP-1R^+^ Tregs (CHD: AUC = 0.600; stenosis: AUC = 0.619). The GLP-1R^+^ Treg proportion was positively correlated with anti-inflammatory IL-35 (r = 0.185, *p* = 0.016) and inversely correlated with IL-4 (r = –0.150, *p* = 0.047).

**Conclusion:**

Reduced Treg frequency and impaired GLP-1R expression on Tregs are independently associated with CHD susceptibility and stenosis progression, potentially mediated by dysregulation of inflammatory cytokines. The GLP-1R pathway in Tregs represents a novel immunomodulatory target for therapeutic intervention in CHD.

## 1. Introduction

Coronary atherosclerotic heart disease (CHD), a chronic inflammatory disorder with autoimmune underpinnings [1], is now widely conceptualized as a pathology driven by dysregulated immune-inflammatory crosstalk. Accumulating evidence has solidified the centrality of inflammatory and immune dysregulation in CHD pathogenesis [2, 3], establishing immunomodulation as a transformative therapeutic frontier in cardiovascular medicine. Regulatory T cells (Tregs) mediate cardioprotective effects in CHD through dual mechanisms: suppressing excessive immune-inflammatory responses [4, 5, 6] facilitating cardiovascular tissue repair [7]. Notably, Tregs are preferentially enriched in atherosclerotic (As) plaques, where they critically mediate inflammation resolution and plaque destabilization—processes indispensable for As regression [8]. Experimental models have revealed that antibody-driven Treg depletion in mice completely negates intensive lipid-lowering therapy-induced plaque regression, abolishing both inflammatory resolution and structural plaque stabilization [9, 10]. Studies have demonstrated that modulating the abundance and functional activity of Tregs effectively suppresses systemic inflammatory responses and inhibits immune cell activation, thereby attenuating the progression of As lesions [11, 12, 13]. However, direct clinical translation of Treg-targeted strategies into CHD therapeutics still faces multiple unresolved challenges.

The glucagon-like peptide-1 receptor (GLP-1R) pathway, a highly scrutinized therapeutic target for type 2 diabetes and obesity [14, 15, 16], exerts multifunctional cardiometabolic benefits through its activation. Specifically, GLP-1R agonism reprograms cardiomyocyte glucose metabolism, attenuates the oxidative stress burden, and suppresses apoptosis signaling, collectively reducing the myocardial infarction area and enhancing cardiac functional recovery. These mechanisms underpin its glycemic control [17], cardioprotective actions (via endothelial stabilization, infarct size limitation, and ventricular remodeling mitigation) [18, 19, 20], and immune-modulatory effects [21, 22]. Recent cutting-edge investigations have revealed that GLP-1R is most highly expressed in induced Tregs, while retaining robust functional integrity in mediating immunomodulatory responses [23]. Preclinical murine models have demonstrated that pharmacological activation of the GLP-1R pathway potentiates the anti-inflammatory efficacy of Tregs through quantitative expansion of Treg populations [24]. However, the pathophysiological relevance of GLP-1R signaling in human Tregs and its mechanistic crosstalk with CHD pathogenesis remain unresolved. In this study, we leveraged multiparametric flow cytometry to quantitatively assess the abundance of Tregs and their glucagon-like GLP-1R expression profiles, coupled with correlative analyses, to investigate their associations with CHD susceptibility and coronary artery stenosis severity. Our findings provide novel immunomodulatory insights into CHD pathogenesis and establish a preclinical evidence base for future therapeutic strategies targeting the Treg-GLP-1R axis to mitigate atherosclerotic cardiovascular complications.

## 2. Materials and Methods

### 2.1 Study Design and Ethical Compliance

This single-center observational study consecutively enrolled 200 patients who presented with chest pain at Dongfeng General Hospital between September 2023 and May 2024. The study protocol was approved by the Institutional Review Board of Sinopharm Dongfeng General Hospital, Hubei University of Medicine, Shiyan, China in compliance with the Declaration of Helsinki. (Approval No. LW-2024-049). Written informed consent was obtained from all participants prior to enrollment.

### 2.2 Participant Stratification

Patients were classified into two groups based on quantitative coronary angiography (QCA): the CHD group (n = 130), with ≥50% luminal stenosis in ≥1 major epicardial artery (left main, LAD, LCx, RCA), was further stratified into chronic coronary syndrome (CCS) and acute coronary syndrome (ACS) per ESC guidelines. The non-CHD group (n = 70) had <50% stenosis in all major arteries and no history of coronary revascularization. The CHD group was further stratified into low-risk and high-risk subgroups based on the median Gensini score.

The exclusion criteria were as follows: (1) metabolic disorders, such as secondary diabetes mellitus or gestational diabetes mellitus; (2) pharmacological interference, such as current or recent use of systemic hormonal therapies, including thyroid hormone replacement or glucocorticoids, which may interfere with immune-metabolic homeostasis; (3) immune dysregulation, such as a history of established autoimmune/inflammatory disorders (including but not limited to rheumatoid arthritis); (4) organ dysfunction, such as severe cardiac insufficiency (left ventricular ejection fraction (LVEF)<40%), decompensated hepatic cirrhosis (Child-Pugh C), or advanced chronic kidney disease (estimated glomerular filtration rate(eGFR) <30 mL/min/1.73 m^2^); (5) oncological conditions, such as active malignancy; and (6) acute systemic illnesses, such as hospitalization-requiring infections and trauma (excluding index myocardial infarction events).

### 2.3 Coronary Angiography and Gensini Scoring

All participants underwent diagnostic coronary angiography using standard techniques. The severity of coronary artery stenosis was quantified using the Gensini scoring system (based on the 2019 guidelines [25]), which accounts for both the degree of luminal narrowing and the anatomical significance of the affected vessels. The Gensini score was calculated as follows: Gensini score = lesion 1 (severity score × weighting factor) + lesion 2 (severity score × weighting factor) + ⋯ + lesion n (severity score × weighting factor). Severity scores (1, 2, 4, 8, 16, and 32) were assigned based on the relative reduction in coronary luminal diameter (25%, 25% ≤ diameter < 50%, 50% ≤ diameter < 75%, 75% ≤ diameter < 90%, 90% ≤ diameter < 99%, and ≥99%, respectively). Weighting factors were applied according to the anatomical location of the stenosis: left main (LM, ×5); proximal (×2.5), mid (×1.5), and distal (×1) segments of the left anterior descending artery (LAD); first diagonal branch (×1) and second diagonal branch (×0.5); proximal (×2.5), mid (×1), and distal (×1) segments of the left circumflex artery (LCX); proximal (×1), mid (×1), and distal (×1) segments of the right coronary artery (RCA); obtuse marginal branch (×1); posterior descending artery (×1); and posterolateral branch (×0.5). Based on the median Gensini score, the coronary heart disease (CHD) group was further stratified into low-risk and high-risk subgroups.

### 2.4 Blood Sampling and Processing

Standardized Blood Collection: Peripheral venous blood (7 mL) was collected from all participants immediately prior to coronary angiography via sterile venipuncture of the radial vein after an overnight fast (≥12 hours). Blood samples were drawn into pre-chilled EDTA vacutainers and maintained at 4 °C throughout processing to preserve cellular integrity and cytokine stability.

Sample Processing Workflow: 2 mL of whole blood was centrifuged at 400 ×g for 10 min (room temperature) within 4 hours post-collection. Plasma aliquots were stored at –80 °C until cytokine profiling (avoiding freeze-thaw cycles). The remaining 5 mL of blood was layered onto human lymphocyte separation medium (Solarbio, #P8610) and centrifuged at 800 ×g for 30 min (18–22 °C). The buffy coat layer (1–2 mm thick, opalescent) was aseptically aspirated using sterile polypropylene transfer pipettes (Thermo Fisher) and transferred into pre-labeled 5 mL flow cytometry tubes. Peripheral blood mononuclear cells (PBMCs) were washed twice at 300 ×g for 10 min at 4 °C with phosphate buffered saline (PBS) (Beyotime). The final cell pellets were resuspended in PBS and immediately subjected to flow cytometry analysis (2 hours post-isolation) to preserve surface epitope integrity.

### 2.5 Flow Cytometric Analysis of Tregs, GLP-1R, and Cytokines

Freshly isolated PBMCs were stained with the following antibody cocktail for surface markers (30 min, room temperature): PerCP-conjugated anti-human CD4 (Clone RPA-T4, BioLegend, #300528), PE-conjugated anti-human CD25 (Clone BC96, BioLegend, #302606), and Alexa Fluor 488-conjugated anti-human GLP-1R (Clone 358903, R&D Systems, #FAB6292G). Intracellular FoxP3-stained cells were fixed/permeabilized using the FoxP3/Transcription Factor Staining Buffer Set (BioLegend, #420801) and stained with Alexa Fluor 647-conjugated anti-FoxP3 (Clone 236A/E7, BioLegend, #320214) for 45 min at room temperature. Cytokine levels interleukin-2 (IL-2), interleukin-4 (IL-4), interleukin-6 (IL-6), interleukin-10 (IL-10), interleukin-35 (IL-35), and tumor necrosis factor-alpha (TNF-α)) were analyzed using a flow cytometric multiplex cytokine kit (Bio-Techne, #IS135; 96 tests).

Using NovoExpress software (version1.3.4, Santa, Clara, CA, USA), cells or cytokines are automatically collected and analyzed. Treg cells are defined as CD4^+^CD25^+^Foxp3^+^ T cells. Lymphocyte populations (lymphocytes) are gated based on FSC-SSC, followed by gating for CD4^+^ T cells. From the CD4^+^ T-cell population, CD25^+^Foxp3^+^ Treg cells were identified, and the number of CD4^+^CD25^+^Foxp3^+^ Treg cells relative to that of CD4^+^ T cells was determined. From the CD4^+^CD25^+^Foxp3^+^ Treg cell population, CD4^+^CD25^+^Foxp3^+^ Treg cells expressing GLP-1R were gated, and their percentage was calculated (see **Supplementary Fig. 1**). For the detection of Treg cells and their GLP-1R expression levels, each sample was collected until 10,000 Treg cells or 500 µL of PBMC suspension was obtained to ensure sufficient statistical power for rare population analysis. The termination condition for collecting cytokines was set at capturing 150 microspheres or reaching a sample volume of 100 µL.

### 2.6 Statistical Analysis

For all variables, normality assumptions were checked with the Shapiro-Wilk test. Normally distributed continuous variables are expressed as the means ± standard deviations (SDs); between-group differences are analyzed via the Student’s *t* test (two groups); nonnormally distributed continuous variables are reported as the medians (interquartile ranges, IQRs) and analyzed using the Mann-Whitney U test (two groups); categorical variables are described as frequencies (percentages) and compared via Pearson’s χ^2^ test or Fisher’s exact test (expected cell counts <5). Univariate and multivariate binary logistic regression analyses were used to evaluate odds ratios (ORs) with 95% confidence intervals (95% CIs). The predictive performance of relevant indicators was assessed using receiver operating characteristic (ROC) curves. Spearman correlation analysis was used to examine the relationship between GLP-1R expression levels and cytokines. All statistical analyses were performed using SPSS software (version 23.0, Armonk, NY, USA), with *p* < 0.05 considered statistically significant.

## 3. Results

### 3.1 Baseline Characteristics of Non-CHD and CHD Groups

Demographic and clinical characteristics of the study cohorts are presented in Table 1. Compared with controls, patients with CHD presented a significantly greater prevalence of hypertension (CHD vs. non-CHD: 59.23% vs. 38.57%, χ^2^ = 7.780, *p* = 0.005) and smoking history (43.08% vs. 27.14%, χ^2^ = 4.929, *p* = 0.026). The CHD group was older [median age: 64 (IQR: 57–70) vs. 59 (IQR: 53–65) years, Z = –3.037, *p* = 0.002] and had a larger waist circumference (93.34 ± 9.77 cm vs. 90.13 ± 10.03 cm, t = –2.196, *p* = 0.029). In contrast, no significant differences were observed in sex distribution (male: 66.92% vs. 57.14%, *p* = 0.171), alcohol consumption (22.31% vs. 20.00%, *p* = 0.705), or lipid profiles, including total cholesterol (4.52 ± 0.96 vs. 4.47 ± 1.12 mmol/L, *p* = 0.745), low-density lipoprotein cholesterol (LDL-C) (2.62 ± 0.90 vs. 2.48 ± 0.89 mmol/L, *p* = 0.267), high-density lipoprotein cholesterol (HDL-C) (1.12 ± 0.28 vs. 1.16 ± 0.37 mmol/L, *p* = 0.402), and triglycerides [median: 1.62 (IQR: 1.20–2.19) vs. 1.72 (IQR: 1.01–2.21) mmol/L, *p* = 0.438]. Body mass index (BMI) also showed no group differences (24.94 ± 3.53 vs. 24.47 ± 3.45 kg/m^2^, *p* = 0.363). Notably, high-sensitivity c-reactive protein (hs-CRP) levels tended to be higher in the CHD group than in the control group but did not reach statistical significance [median: 1.33 (IQR: 0.62–3.71) vs. 0.97 (0.53–2.49) mg/L, *p* = 0.218]. These results underscore hypertension, smoking, advanced age, and central adiposity as key distinguishing factors in CHD patients, whereas traditional lipid markers and systemic inflammation showed limited discriminative capacity in this cohort.

**Table 1.** **Comparison of baseline data between the non-CHD group and the CHD group**.

Variable	Non-CHD group (n = 70)	CHD group (n = 130)	χ^2^/t/Z	*p* value
Male sex, n (%)	40 (57.14%)	87 (66.92%)	1.878	0.171
Smoking history, n (%)	19 (27.14%)	56 (43.08%)	4.929	0.026
Alcohol consumption, n (%)	14 (20.00%)	29 (22.31%)	0.144	0.705
Hypertension, n (%)	27 (38.57%)	77 (59.23%)	7.780	0.005
Age, years	59 (53, 65)	64 (57, 70)	–3.037	0.002
Waist circumference, cm	90.13 ± 10.03	93.34 ± 9.77	–2.196	0.029
Body mass index (BMI), kg/m^2^	24.47 ± 3.45	24.94 ± 3.53	–0.911	0.363
hs-CRP, mg/L	0.97 (0.53, 2.49)	1.33 (0.62, 3.71)	–1.231	0.218
Total cholesterol, mmol/L	4.47 ± 1.12	4.52 ± 0.96	–0.325	0.745
Triglycerides, mmol/L	1.72 (1.01, 2.21)	1.62 (1.20, 2.19)	–0.797	0.438
HDL-C, mmol/L	1.16 ± 0.37	1.12 ± 0.28	0.842	0.402
LDL-C, mmol/L	2.48 ± 0.89	2.62 ± 0.90	–1.114	0.267

HDL-C, high-density lipoprotein cholesterol; LDL-C, low-density lipoprotein cholesterol; hs-CRP, high-sensitivity c-reactive protein.

### 3.2 Reduced Frequency of Tregs and GLP-1R^+^ Tregs in the CHD Group

**Fig. 1.**
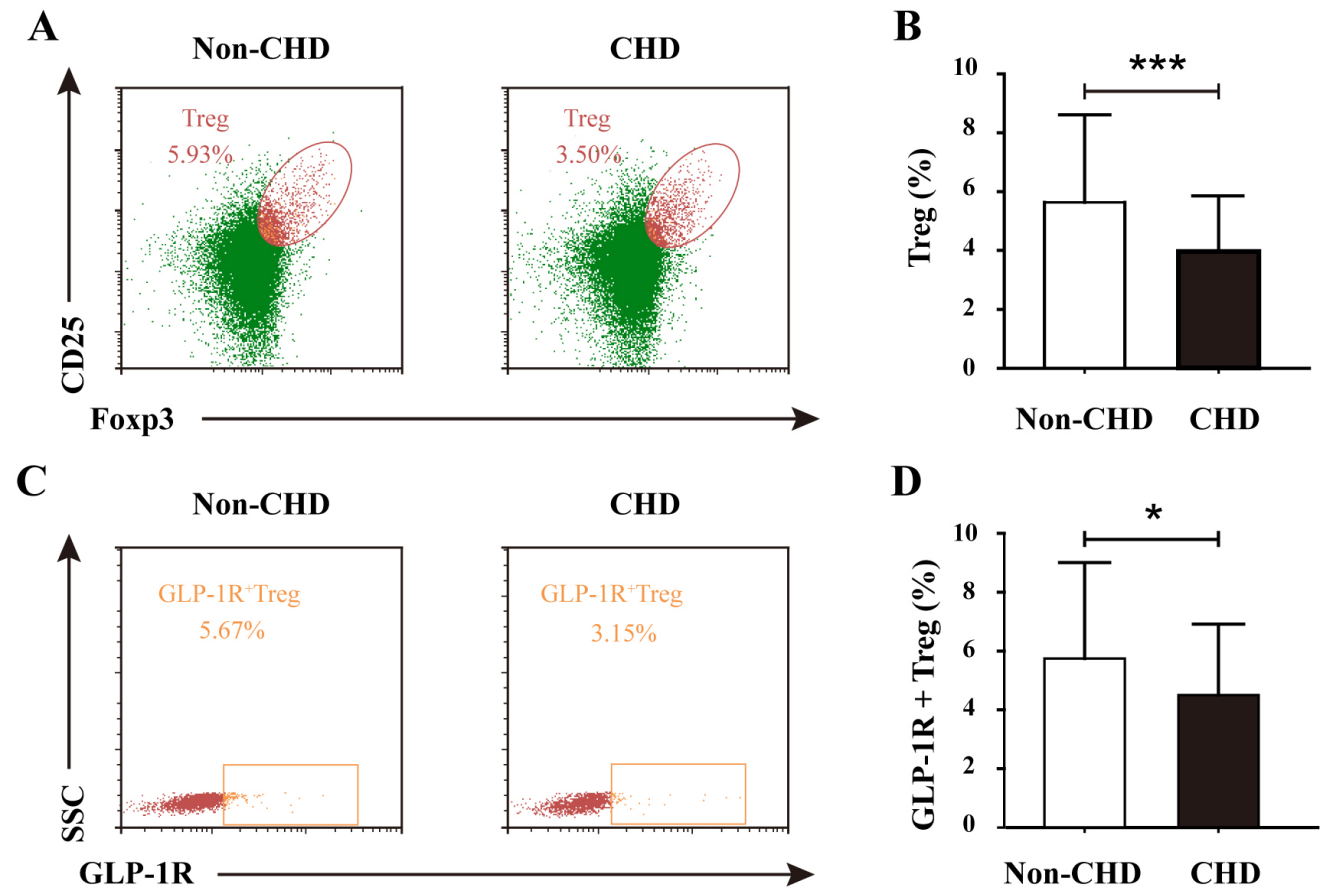
**Comparative analysis of Treg frequency and GLP-1R^+^ Treg subsets in non-CHD and CHD groups**. (A) Representative flow cytometry plots gating CD4^+^CD25^+^FoxP3^+^ Tregs in the non-CHD and CHD groups. (B) Quantification of Treg frequency (% of CD4^+^ T cells) demonstrating significant reduction in the CHD groups (***p < 0.001, unpaired Student’s *t* test). (C) Flow plots illustrating GLP-1R^+^ Tregs within the Treg population. (D) The proportion of GLP-1R^+^ Tregs (% of total Tregs) was markedly lower in the CHD group than in the non-CHD group (*p < 0.05, unpaired *t* test). The data are expressed as the means ± standard deviations (SDs) (non-CHD, n = 70; CHD, n = 130). GLP-1R, glucagon-like peptide-1 receptor; CHD, coronary heart disease.

Comparative immunophenotypic analysis demonstrated significant alterations in Treg cell dynamics among CHD patients. The frequency of circulating regulatory T cells (Tregs, CD4^+^CD25^+^FoxP3^+^) was substantially reduced in the CHD cohort compared with that in the non-CHD control group (Fig. 1A). In the non-CHD group, the proportion of Treg cells was 5.93%, whereas in the CHD group, it was 3.50%. The difference was highly statistically significant (Fig. 1B). This depletion was further accentuated in CHD patients with high-risk coronary stenosis (Gensini score ≥ median), who exhibited a significant reduction relative to controls (*p* < 0.001).

Notably, the immunoregulatory defect extended to the subset of Tregs expressing glucagon-like peptide-1 receptor (GLP-1R^+^ Tregs). In the non-CHD group, the proportion of GLP-1R^+^ Treg double-positive cells was 5.67% compared with 3.15% in the CHD group (Fig. 1C). The proportion of GLP-1R^+^ Tregs was significantly greater in the non-CHD group than in the CHD group (Fig. 1D). Stratification by stenosis severity revealed a progressive decline in GLP-1R^+^ Treg representation, with high-risk patients displaying significantly lower levels than their low-risk counterparts (*p* < 0.05).

These findings collectively suggest a dual defect in both Treg abundance and functional GLP-1R-mediated immunomodulatory capacity, potentially contributing to sustained inflammatory activation in coronary atherosclerosis.

### 3.3 Association Between the Frequency of Treg and GLP-1R^+^Treg and CHD Risk

Univariate logistic regression revealed a significant inverse correlation between Treg frequency and CHD risk (OR = 0.749, 95% CI: 0.657–0.854; *p* < 0.001). This association persisted after multivariate adjustment for sex, smoking history, and lipid profiles (adjusted OR = 0.752, 95% CI: 0.645–0.877, *p* < 0.001; **Supplementary Table 1**), indicating that Treg depletion is an independent protective factor against CHD. ROC curve analysis demonstrated a certain degree of discriminative capacity of Treg frequency for CHD risk, with an AUC of 0.663 (95% CI: 0.581–0.746) (**Supplementary Fig. 2A**). A restricted cubic spline (RCS) model further delineated a nonlinear dose-response relationship, wherein CHD risk decreased progressively with increasing Treg frequency (**Supplementary Fig. 2B**).

Univariate logistic regression revealed that a reduced GLP-1R^+^ Treg frequency (% of total Tregs) was significantly associated with elevated CHD risk (OR = 0.852, 95% CI: 0.765–0.948, *p* = 0.003). Additional risk factors included smoking history (OR = 2.031, 95% CI: 1.081–3.817, *p* = 0.028), hypertension (OR = 2.314, 95% CI: 1.276–4.195, *p* = 0.006), advanced age (OR = 1.050, 95% CI: 1.017–1.084, *p* = 0.003), and increased waist circumference (OR = 1.035, 95% CI: 1.003–1.069, *p* = 0.031). After adjusting for sex, lipid profiles, and the aforementioned covariates, the inverse correlation between GLP-1R^+^ Treg frequency and CHD risk remained robust (adjusted OR = 0.859, 95% CI: 0.762–0.969, *p* = 0.013) (Table 2), confirming its role as an independent protective factor against coronary atherosclerosis.

**Table 2.** **Logistic regression analysis of GLP-1R^+^ Treg frequency and CHD risk**.

Variable	Univariate analysis			Multifactor analysis		
	β	OR (95% CI)	*p*	β	OR (95% CI)	*p*
GLP-1R^+^ Treg	–0.161	0.852 (0.765, 0.948)	0.003	–0.152	0.859 (0.762, 0.969)	0.013
Sex	–0.417	0.659 (0.362, 1.198)	0.172	0.044	1.045 (0.428, 2.554)	0.923
Smoking history	0.709	2.031 (1.081, 3.817)	0.028	0.934	2.546 (1.022, 6.337)	0.045
Alcohol history	0.138	1.149 (0.561, 2.351)	0.705	–0.301	0.740 (0.292, 1.875)	0.525
Hypertension	0.839	2.314 (1.276, 4.195)	0.006	0.867	2.380 (1.194, 4.745)	0.014
Age	0.049	1.050 (1.017, 1.084)	0.003	0.066	1.069 (1.027, 1.112)	0.001
Waist circumference	0.035	1.035 (1.003, 1.069)	0.031	0.025	1.025 (0.978, 1.074)	0.298
Body mass index	0.039	1.040 (0.956, 1.132)	0.362	0.029	1.029 (0.902, 1.174)	0.669
hs-CRP	0.008	1.008 (0.979, 1.038)	0.605	0.001	1.000 (0.969, 1.032)	0.989
Total cholesterol	0.048	1.049 (0.787, 1.398)	0.744	–0.690	0.501 (0.223, 1.129)	0.095
Triglycerides	0.168	1.183 (0.854, 1.640)	0.312	0.288	1.334 (0.852, 2.088)	0.208
HDL-C	–0.434	0.648 (0.256, 1.641)	0.360	0.531	1.701 (0.428, 6.761)	0.451
LDL-C	0.188	1.206 (0.867, 1.679)	0.266	0.965	2.624 (1.085, 6.345)	0.032

OR, odds ratios.

**Fig. 2.**
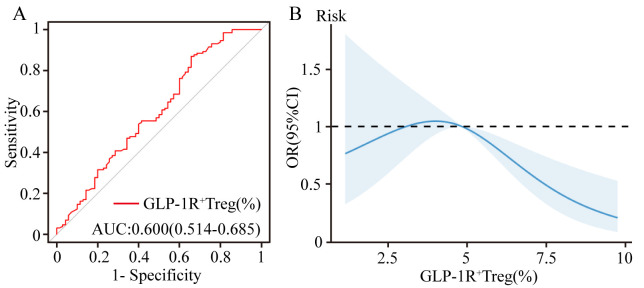
**Predictive and dose-response relationships of GLP-1R^+^ Tregs in CHD risk assessment**. (A) Receiver operating characteristic (ROC) curve analysis evaluating the discriminative capacity of the GLP-1R^+^ Treg frequency (% of total Tregs) for CHD risk stratification (AUC: 0.600). (B) Restricted cubic spline (RCS) model illustrating the nonlinear inverse association between GLP-1R^+^ Treg frequency and CHD risk.

ROC curve analysis demonstrated that GLP-1R^+^ Treg frequency (% of total Tregs) exhibited modest discriminative capacity for differentiating CHD patients from non-CHD controls, with an AUC of 0.600 (95% CI: 0.514–0.685, *p* = 0.032) (Fig. 2A). While the AUC value suggests limited standalone diagnostic utility, its statistical significance (*p* < 0.05) implies potential relevance in composite risk models. Furthermore, the RCS model revealed a nonlinear inverse relationship between GLP-1R^+^ Treg frequency and CHD risk (Fig. 2B). Elevated GLP-1R+ Treg levels were associated with a reduction in CHD risk, reinforcing the protective role of GLP-1R signaling in Treg-mediated immunomodulation.

### 3.4 Comparison and Balance Analysis of Baseline Characteristics Between Low-Risk and High-Risk CHD Patients Stratified by Gensini Score

Patients with CHD were stratified into low-risk (n = 68) and high-risk (n = 62) subgroups based on the median Gensini score. Baseline characteristics of both groups are presented in Table 3. No statistically significant differences were observed between the low-risk and high-risk groups across all evaluated parameters (all *p* > 0.05). Specifically, demographic and clinical factors such as male sex (61.76% vs. 72.58%; *p* = 0.190), smoking history (39.71% vs. 46.77%; *p* = 0.416), and prevalence of hypertension (61.76% vs. 56.45%; *p* = 0.538) showed comparable distributions. Similarly, metabolic markers, including lipid profiles (total cholesterol: 4.25 vs. 4.54 mmol/L, *p* = 0.366; LDL-C: 2.52 vs. 2.73 mmol/L, *p* = 0.188) and inflammatory indicators (hs-CRP: 1.13 vs. 1.51 mg/L, *p* = 0.185), did not differ significantly between groups. Although alcohol consumption (16.18% vs. 29.03%; *p* = 0.079) and waist circumference (*p* = 0.073) demonstrated trends toward higher values in the high-risk group, these differences did not reach statistical significance. These findings suggest that baseline characteristics were well balanced between the two subgroups, supporting the validity of risk stratification based on the Gensini score in this cohort.

**Table 3.** **Comparison of baseline data between low-risk and high-risk CHD groups**.

Variable	Low-Risk CHD (n = 68)	High-Risk CHD (n = 62)	χ^2^/t/Z	*p*
Male sex, n (%)	42 (61.76%)	45 (72.58%)	1.714	0.190
Smoking history, n (%)	27 (39.71%)	29 (46.77%)	0.661	0.416
Alcohol consumption, n (%)	11 (16.18%)	18 (29.03%)	3.093	0.079
Hypertension, n (%)	42 (61.76%)	35 (56.45%)	0.379	0.538
Age, years	62.71 ± 8.31	65.26 ± 10.30	2.919	0.121
Waist circumference, cm	92.00 (85.00, 98.75)	93.50 (85.00, 101.25)	–1.790	0.073
Body mass index (BMI), kg/m^2^	24.80 ± 2.88	25.09 ± 4.14	5.626	0.642
hs-CRP, mg/L	1.13 (0.61, 2.94)	1.51 (0.64, 4.18)	–1.325	0.185
Total cholesterol, mmol/L	4.25 (3.67, 5.21)	4.54 (3.70, 5.29)	–0.905	0.366
Triglycerides, mmol/L	1.60 (1.13, 2.05)	1.80 (1.23, 2.42)	–1.656	0.098
HDL-C, mmol/L	1.15 (0.91, 1.33)	1.09 (0.87, 1.27)	–1.042	0.297
LDL-C, mmol/L	2.52 ± 0.81	2.73 ± 0.99	1.653	0.188

### 3.5 Comparison of Treg Cell Proportions and GLP-1R Levels Between Low-Risk and High-Risk CHD Groups

**Fig. 3.**
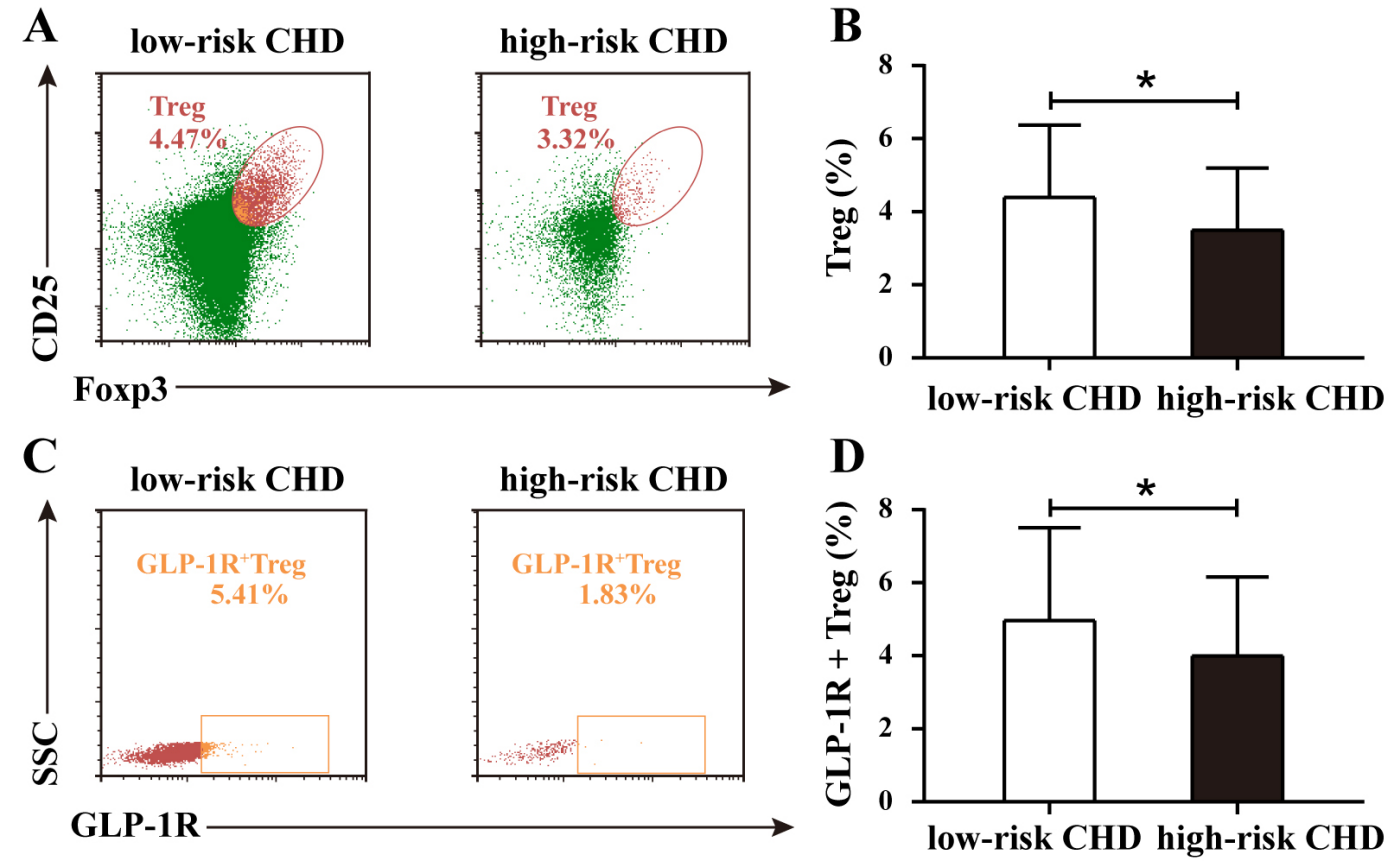
**Proportions of Treg and GLP-1R^+^ Treg cells in low-risk and high-risk CHD groups**. (A) Representative flow cytometry plots of Treg proportions in low-risk and high-risk CHD groups. (B) Comparative analysis of Treg proportions between the low-risk CHD group and high-risk CHD group. (C) Representative flow cytometry plots of GLP-1R^+^ Treg proportions in low-risk and high-risk CHD groups. (D) Comparative analysis of GLP-1R^+^ Treg cell proportions between groups. Data are expressed as the means ± SDs and were analyzed using the Student’s *t* test (*p < 0.05).

Flow cytometry was used to analyze the proportions of regulatory T cells (Tregs) and GLP-1R^+^ Tregs in the low-risk and high-risk CHD groups. As shown in Fig. 3A, the proportion of Treg cells, determined by the coexpression of CD25 and Foxp3, was 4.47% in the low-risk CHD group and 3.32% in the high-risk CHD group. As shown in Fig. 3C, based on the expression of SSC and GLP-1R, the proportion of GLP-1R^+^ Treg cells was 5.41% in the low-risk CHD group and 1.83% in the high-risk CHD group. Statistical analysis was conducted to evaluate the differences in the proportions of Treg and GLP-1R^+^ Treg cells between the two groups. The results revealed a significant difference in the proportion of Treg cells between the low-risk CHD group and high-risk CHD group (*p* < 0.05; Fig. 3B), and the proportion of GLP-1R^+^ Treg cells also exhibited a significant inter-group difference (*p* < 0.05; Fig. 3D). These results indicate that obvious differences exist in the immune cell populations related to regulatory T cells between the low-risk CHD group and high-risk CHD group, which may have an impact on the pathophysiology of CHD.

### 3.6 Correlation of Treg and GLP-1R^+^ Treg Frequencies and Coronary Stenosis Severity

Univariate analysis identified that Treg cell depletion was a significant predictor of advanced stenosis (OR: 0.762, *p* = 0.009). Multivariate adjustment for demographic and metabolic confounders preserved this association (adjusted OR: 0.760, *p* = 0.021) (**Supplementary Table 2**), highlighting the independent protective role of Treg cells. ROC analysis supported their discriminatory capacity (AUC: 0.635, 95% CI: 0.539–0.731) (**Supplementary Fig. 3A**), whereas RCS modeling revealed a nonlinear inverse relationship between Treg proportions and stenosis severity (**Supplementary Fig. 3B**), underscoring their therapeutic potential in halting atherosclerosis.

**Fig. 4.**
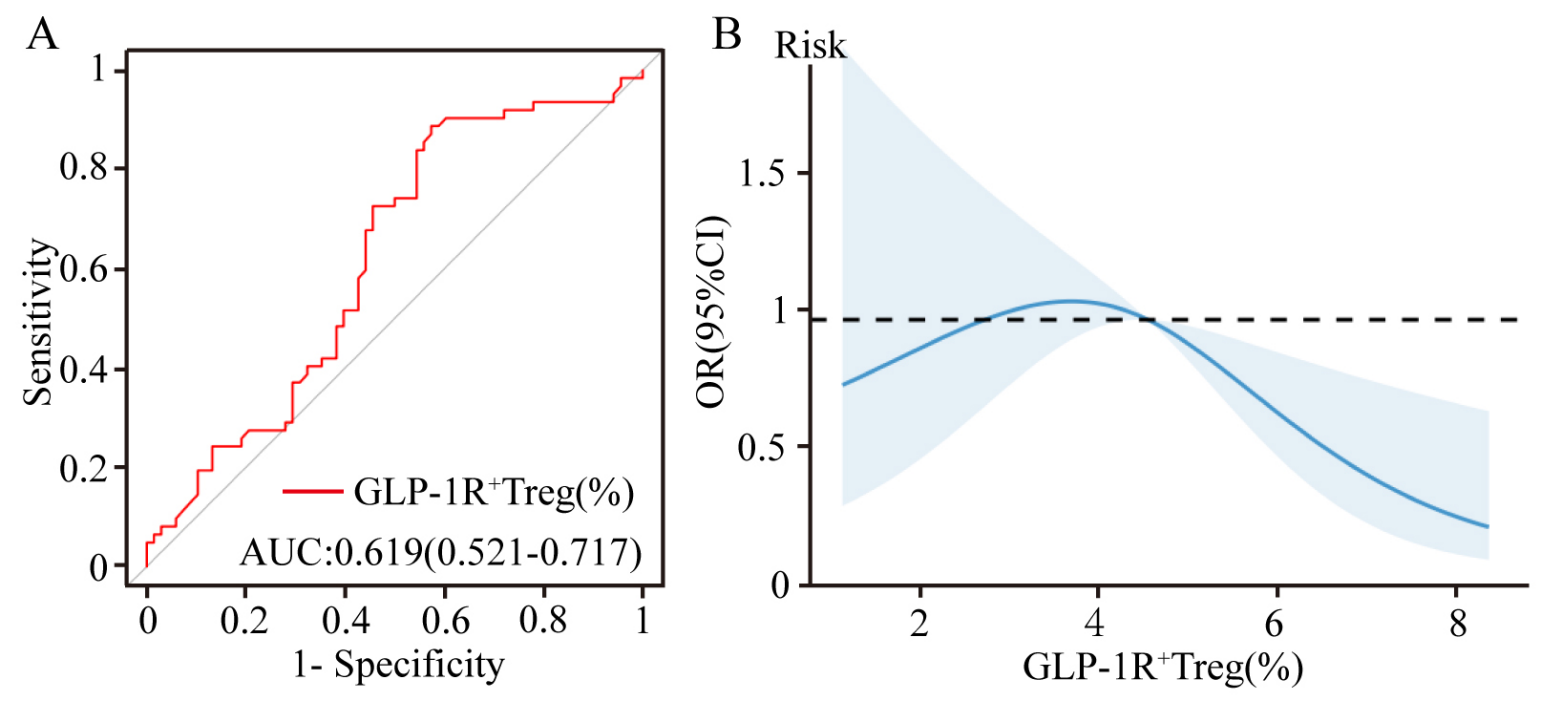
**Predictive and dose-response relationship of GLP-1R^+^ Treg cells proportions with coronary stenosis severity**. (A) ROC curve evaluating GLP-1R^+^ Treg cell proportions in predicting stenosis severity (AUC: 0.619). (B) Restricted cubic spline plot illustrating the inverse association between GLP-1R^+^ Treg proportions and stenosis severity. ROC, receiver operating characteristic; AUC, area under the curve.

Univariate logistic regression revealed a protective role of GLP-1R^+^ Treg cells against coronary stenosis (OR: 0.840, *p* = 0.023). Multivariate adjustment for demographic and clinical confounders preserved this association (adjusted OR: 0.840, *p* = 0.040), underscoring GLP-1R signaling as a potential modulator of plaque stability. For detailed regression coefficients, refer to Table 4. ROC analysis demonstrated that the proportion of GLP-1R^+^ Tregs moderately predicted stenosis severity (AUC: 0.619, 95% CI: 0.521–0.717; Fig. 4A). RCS modeling further revealed a dose-dependent protective effect: each incremental increase in GLP-1R^+^ Treg proportions was correlated with attenuated stenosis severity (Fig. 4B), implicating GLP-1R signaling as a therapeutic target to decelerate coronary atherosclerosis.

**Table 4.** **Logistic regression analysis of the proportion of GLP-1R^+^ Treg predicting coronary artery stenosis severity in CHD**.

Variable	Univariate analysis			Multifactor analysis		
	β	OR (95% CI)	*p*	β	OR (95% CI)	*p*
GLP-1R^+^ Treg	–0.174	0.840 (0.724, 0.976)	0.023	–0.174	0.840 (0.712, 0.992)	0.040
Sex	–0.494	0.610 (0.291, 1.282)	0.172	–0.362	0.697 (0.240, 2.026)	0.507
Smoking history	0.289	1.334 (0.665, 2.677)	0.417	–0.136	0.872 (0.334, 2.282)	0.781
Alcohol history	0.751	2.120 (0.909, 4.945)	0.082	0.484	1.622 (0.593, 4.434)	0.346
Hypertension	–0.220	0.802 (0.398, 1.617)	0.538	–0.362	0.696 (0.310, 1.564)	0.381
Age	0.030	1.030 (0.992, 1.070)	0.122	0.062	1.064 (1.012, 1.119)	0.015
Waist circumference	0.019	1.019 (0.983, 1.056)	0.307	–0.001	0.999 (0.945, 1.056)	0.967
Body mass index	0.024	1.024 (0.928, 1.130)	0.634	0.068	1.070 (0.922, 1.241)	0.371
hs-CRP	0.033	1.034 (0.969, 1.103)	0.315	0.032	1.032 (0.959, 1.110)	0.398
Total cholesterol	0.175	1.191 (0.830, 1.710)	0.343	–0.294	0.745 (0.225, 2.471)	0.631
Triglycerides	0.316	1.371 (0.941, 1.997)	0.100	0.443	1.557 (0.896, 2.705)	0.116
HDL-C	–0.834	0.434 (0.121, 1.566)	0.203	–0.161	0.851 (0.155, 4.690)	0.853
LDL-C	0.261	1.298 (0.880, 1.914)	0.188	0.576	1.779 (0.531, 5.957)	0.350

### 3.7 GLP-1R^+^ Treg Cells Coordinate Anti-Inflammatory Cytokine Networks

**Fig. 5.**
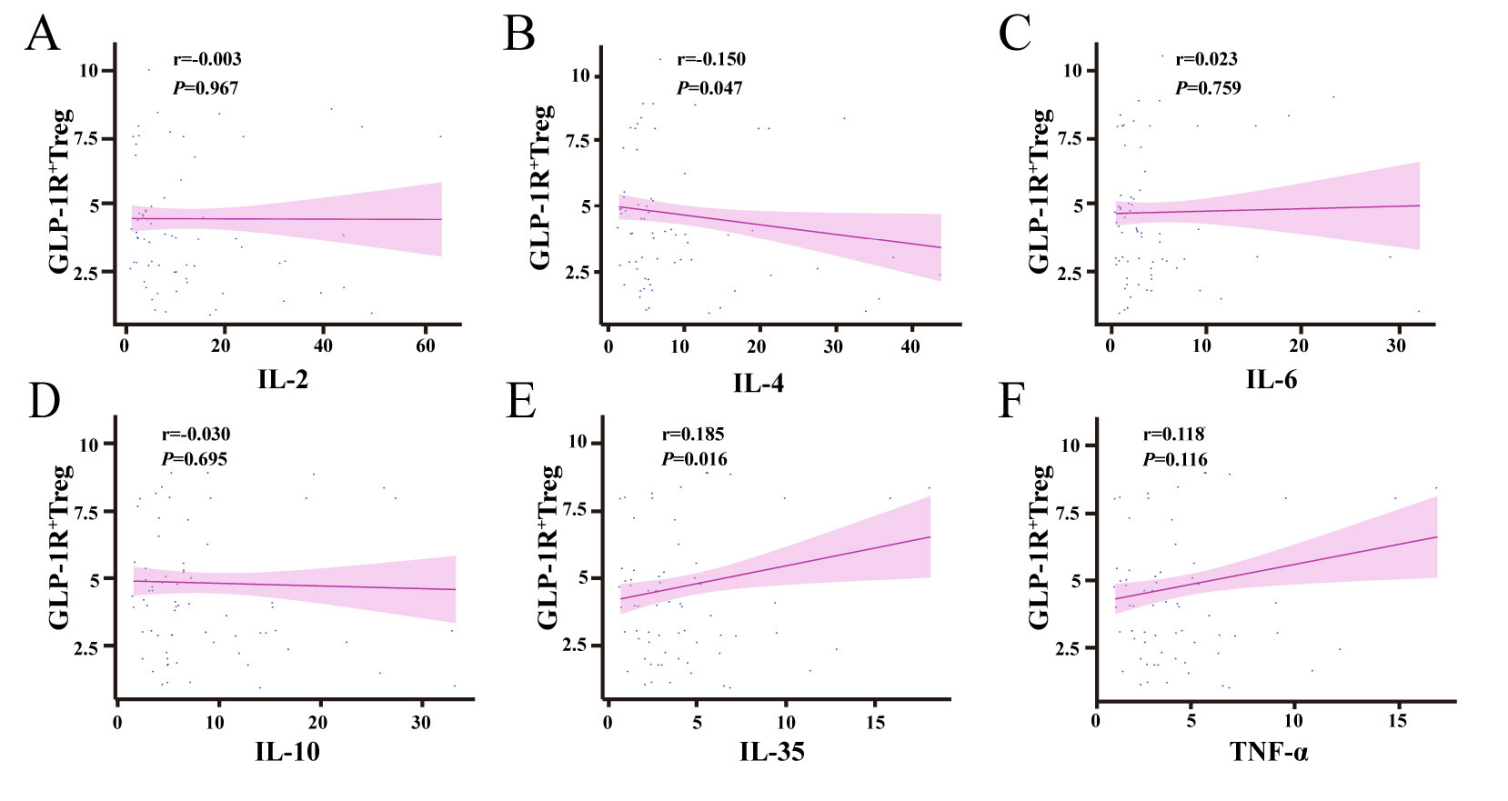
**Correlation analysis between GLP-1R^+^ Treg cell proportions and cytokine levels**. (A) No significant correlation was observed between GLP-1R^+^ Treg proportions and IL-2 levels (*p* > 0.05). (B) GLP-1R^+^ Treg proportions were negatively correlated with IL-4 levels (r = –0.150, *p* = 0.047). (C) No association was found between GLP-1R^+^ Treg proportions and IL-6 levels (*p* > 0.05). (D) IL-10 levels were not significantly correlated with GLP-1R^+^ Treg proportions (*p* > 0.05). (E) GLP-1R^+^ Treg proportions were positively correlated with IL-35 levels (r = 0.185, *p* = 0.016). (F) TNF-α levels were not associated with GLP-1R^+^ Treg proportions (*p* > 0.05).

To investigate the regulatory role of GLP-1R^+^ Treg cells in cytokine networks, correlation analyses were performed between their proportions and key cytokine levels in the study cohort. A statistically significant negative correlation was observed between GLP-1R^+^ Treg cells and interleukin-4 (IL-4) levels (r = –0.150, *p* = 0.047; Fig. 5B), whereas a significant positive correlation was detected with interleukin-35 (IL-35) levels (r = 0.185, *p* = 0.016; Fig. 5E). No significant associations were found with other cytokines, including interleukin-2 (IL-2; r = –0.003, *p* = 0.967; Fig. 5A), interleukin-6 (IL-6; r = 0.023, *p* = 0.759; Fig. 5C), interleukin-10 (IL-10; r = 0.030, *p* = 0.695; Fig. 5D), or tumor necrosis factor-alpha (TNF-α; r = 0.118, *p* = 0.116; Fig. 5F). These results indicate that GLP-1R^+^ Treg cells selectively modulate IL-4 and IL-35, potentially suppressing IL-4 while enhancing IL-35 without broad effects on other cytokines. This differential regulation may contribute to immune homeostasis in CHD, underscoring the nuanced role of GLP-1R signaling in Treg-mediated immune responses.

## 4. Discussion

To our knowledge, this study is the first to demonstrate that reduced proportions of Treg cells and diminished GLP-1R expression on Treg cells are independently associated with an increased risk of CHD and greater coronary artery stenosis severity. Individuals with lower Treg cell levels and impaired GLP-1R signaling exhibited a significantly elevated risk of CHD incidence and accelerated plaque progression, highlighting their dual role as predictive biomarkers and pathogenic contributors.

Consistent with prior studies in chronic coronary syndrome [22], our findings revealed a significant reduction in circulating Treg cell proportions among CHD patients. This Treg deficiency was inversely associated with CHD risk, aligning with clinical evidence linking low Treg levels to heightened susceptibility to acute coronary syndrome [26]. Preclinical studies further corroborate these observations: Treg depletion exacerbates atherogenesis in hypercholesterolemic mice [27], whereas Treg expansion attenuates atherosclerotic plaque burden [28] and even induces plaque regression [29]. Collectively, these data support the hypothesis that elevated Treg cell levels may mitigate CHD risk by slowing atherosclerotic progression through immunomodulatory mechanisms.

Our study revealed a significant reduction in circulating GLP-1R^+^ Treg cell proportions among CHD patients compared with those of non-CHD controls, with lower GLP-1R^+^ Treg levels correlated inversely with CHD risk. Emerging evidence suggests that GLP-1R signaling plays a pivotal role in immune regulation [30]. Preclinical studies have demonstrated that GLP-1 receptor agonists (GLP-1RAs) enhance Treg cell frequency in obese diabetic mice [16] and high-fat diet-induced models [24]. Notably, GlP-1R^-⁣/-^ mice exhibit markedly reduced Treg proportions in lymph nodes and peripheral blood compared those in wild-type (GlP-1R^+⁣/+^) littermates, underscoring the necessity of intact GLP-1R signaling for Treg homeostasis and immunosuppressive function [21]. These findings collectively imply that elevated GLP-1R expression on Treg cells may confer protection against CHD by modulating both Treg abundance and functional efficacy. Both univariate and multivariate logistic regression analyses demonstrated that reduced proportions of Treg cells and diminished GLP-1R expression on Treg cells were independently associated with increased coronary artery stenosis severity in CHD patients. ROC curve analysis further confirmed the moderate discriminatory capacity of Treg proportions and GLP-1R^+^ Treg levels for stenosis severity. RCS models revealed a nonlinear inverse dose-response relationship: individuals with lower Treg proportions or impaired GLP-1R signaling exhibited significantly aggravated stenosis. Based on these findings, it can be hypothesized that GLP-1RA may play a potential role in CHD by partially activating GLP-1R on the surface of Treg cells, which in turn affects the number and function of Treg cells. Therefore, more in-depth studies are necessary to verify whether GLP-1R^+^ Tregs may serve as modifiable immunotherapeutic targets for CHD.

The progressive decline in Treg cell levels during coronary plaque progression may arise from multiple mechanisms: (1) Impaired Treg Generation: Dysfunctional thymic Treg production and metabolic reprogramming induced by hyperlipidemia [31]. (2) Enhanced Apoptosis: oxidative stress [32] or epigenetic dysregulation of Foxp3 [33] increased susceptibility to apoptosis [34]. (3) Phenotypic Shifts: Altered immunosuppressive capacity of Tregs in advanced atherosclerosis [11, 35]. Severe coronary stenosis exacerbates myocardial ischemia, triggering inflammatory activation and reactive oxygen species (ROS) overproduction [36]. Conversely, GLP-1R activation counteracts these pathways by suppressing pro-inflammatory cytokines and enhancing antioxidant defenses [20, 37], suggesting a feedback loop where GLP-1R^+^ Treg loss amplifies plaque instability.

Treg cells exert immunosuppressive effects via cell-cell contact and secretion of inhibitory cytokines, including interleukin-10 (IL-10) [38, 39], IL-35 [40], and transforming growth factor-beta (TGF-β) [41, 42]. Our study further revealed that GLP-1R^+^ Treg proportions positively correlated with IL-35 levels and inversely correlated with IL-4 levels, suggesting that GLP-1R activation may enhance Treg immunosuppressive function by rebalancing cytokine networks. GLP-1 receptor agonists (GLP-1RAs), such as semaglutide, reduce aortic plaque burden in Apoe^-⁣/-^ mice by suppressing pro-inflammatory cytokines (e.g., IFN-γ and TNF-α) [43]. Similarly, liraglutide attenuates systemic inflammation in humans by lowering IL-1β and TNF-α levels [44]. Mechanistically, GLP-1R signaling activates cyclic adenosine monophosphate (cAMP), triggering AMP-activated protein kinase (AMPK) pathways to promote Treg proliferation and functional enhancement [45, 46, 47]. In diabetic murine models, GLP-1RAs rescue Treg dysfunction by reducing SP1 O-GlcNAcylation and upregulating NCLX [48]. Additionally, exenatide may promote Treg development via the PI3K/Akt/FoxO1 axis [16], although further validation is required.

This study has several limitations. First, the relatively small sample size may introduce selection bias and limit the generalizability of our findings. Second, the cross-sectional design precludes causal inference between Treg cell proportions, GLP-1R^+^ Treg levels, and CHD risk or stenosis severity. Future large-scale, multicenter prospective cohort studies are warranted to validate these associations. Moreover, the use of single-time-point blood samples may not fully capture the dynamic changes in Treg cell populations and their GLP-1R expression over time. Longitudinal studies with repeated measurements would be valuable to address this limitation. Finally, our analysis focused on circulating Treg cells rather than plaque-infiltrating Treg populations, which may exhibit distinct GLP-1R expression patterns and functional dynamics. Despite these limitations, our study provides important insights into the potential role of Treg cells and GLP-1R signaling in CHD pathogenesis and highlights the need for further investigation in this area.

## 5. Conclusion

This study demonstrates that reduced levels of Treg cells and diminished GLP-1R expression on Treg cells are closely associated with the incidence of CHD and independently correlated with the severity of coronary artery stenosis. The GLP-1R signaling pathway in Treg cells may hold potential as a novel immunomodulatory therapeutic target for CHD, offering a dual strategy to mitigate both metabolic dysfunction and inflammatory plaque progression.

## Availability of Data and Materials

The datasets used and analyzed during the current study are available from the corresponding author on reasonable request.

## Supplementary Material

Supplementary material associated with this article can be found, in the online version, at https://doi.org/10.31083/RCM39927.
