# Supplementary material for: An Association Between GLP-1 Receptor Expression on Regulatory T Cells and the Severity of Coronary Artery Stenosis and Inflammatory Dysregulation in Coronary Heart Disease
Source: Rev Cardiovasc Med. 2025 Sep 25;26(9):39927. doi: 10.31083/RCM39927 (PMC12516772; doi:10.31083/RCM39927)
Supplement: Supplementary file 1 [file 2153-8174-26-9-39927-s1.docx]

**Supplementary Tables and Figures**

**Supplementary Table 1. Logistic Regression Analysis of Treg Frequency and CHD Risk.**

| Variable | Univariate analysis | | | Multifactor analysis | | |
| --- | --- | --- | --- | --- | --- | --- |
|  | β | OR (95%CI) | *P* | β | OR (95%*CI*) | *P* |
| Treg | -0.289 | 0.749(0.657,0.854) | <0.001 | -0.285 | 0.752(0.645,0.877) | <0.001 |
| Sex | -0.417 | 0.659(0.362,1.198) | 0.172 | -0.193 | 0.824(0.331,2.053) | 0.678 |
| Smoking history | 0.709 | 2.031(1.081,3.817) | 0.028 | 0.973 | 2.647(1.043,6.714) | 0.040 |
| Alcohol history | 0.138 | 1.149(0.561,2.351) | 0.705 | -0.295 | 0.745(0.283,1.957) | 0.550 |
| Hypertension | 0.839 | 2.314(1.276,4.195) | 0.006 | 0.977 | 2.657(1.301,5.424) | 0.007 |
| Age | 0.049 | 1.050(1.017,1.084) | 0.003 | 0.059 | 1.061(1.019,1.104) | 0.004 |
| Waist circumference | 0.035 | 1.035(1.003,1.069) | 0.031 | 0.013 | 1.013(0.966,1.063) | 0.588 |
| Body mass index | 0.039 | 1.040(0.956,1.132) | 0.362 | 0.018 | 1.018(0.888,1.167) | 0.801 |
| hs-CRP | 0.008 | 1.008(0.979,1.038) | 0.605 | 0.003 | 1.003(0.965,1.043) | 0.870 |
| Total cholesterol | 0.048 | 1.049(0.787,1.398) | 0.744 | -0.725 | 0.484(0.194,1.212) | 0.121 |
| Triglycerides | 0.168 | 1.183(0.854,1.640) | 0.312 | 0.235 | 1.265(0.788,2.030) | 0.331 |
| HDL-C | -0.434 | 0.648(0.256,1.641) | 0.360 | 0.821 | 2.272(0.525,9.834) | 0.272 |
| LDL-C | 0.188 | 1.206(0.867,1.679) | 0.266 | 0.983 | 2.672(1.006,7.093) | 0.049 |

**Supplementary Table 2. Logistic Regression Analysis of Treg Proportion Predicting.**

**Coronary Artery Stenosis Severity in CHD**

| Variable | Univariate analysis | | | Multifactor analysis | | |
| --- | --- | --- | --- | --- | --- | --- |
|  | β | OR (95%*CI)* | *P* | β | OR (95%*CI*) | *P* |
| Treg | -0.272 | 0.762(0.622,0.935) | 0.009 | -0.274 | 0.760(0.603,0.959) | 0.021 |
| Sex | -0.494 | 0.610(0.291,1.282) | 0.172 | -0.397 | 0.673(0.228,1.984) | 0.472 |
| Smoking history | 0.289 | 1.334(0.665,2.677) | 0.417 | -0.044 | 0.957(0.364,2.514) | 0.929 |
| Alcohol history | 0.751 | 2,120(0.909,4.945) | 0.082 | 0.495 | 1.641(0.592,4.553) | 0.341 |
| Hypertension | -0.220 | 0.802(0.398,1.617) | 0.538 | -0.197 | 0.821(0.359,1.877) | 0.641 |
| Age | 0.030 | 1.030(0.992,1.070) | 0.122 | 0.058 | 1.060(1.009,1.113) | 0.020 |
| Waist circumference | 0.019 | 1.019(0.983,1.056) | 0.019 | -0.013 | 0.987(0.933,1.043) | 0.641 |
| Body mass index | 0.024 | 1.024(0.928,1.130) | 0.634 | 0.072 | 1.075(0.922,1.253) | 0.356 |
| hs-CRP | 0.033 | 1.034(0.969,1.103) | 0.315 | 0.047 | 1.048(0.971,1.132) | 0.230 |
| Total cholesterol | 0.175 | 1.191(0.830,1.710) | 0.343 | -0.437 | 0.646(0.198,2.112) | 0.470 |
| Triglycerides | 0.316 | 1.371(0.941,1.997) | 0.100 | 0.407 | 1.503(0.872,2.590) | 0.142 |
| HDL-C | -0.834 | 0.434(0.121,1.566) | 0.203 | 0.239 | 1.270(0.214,7.536) | 0.793 |
| LDL-C | 0.261 | 1.298(0.880,1.914) | 0.188 | 0.769 | 2.158(0.652,7.139) | 0.208 |

**
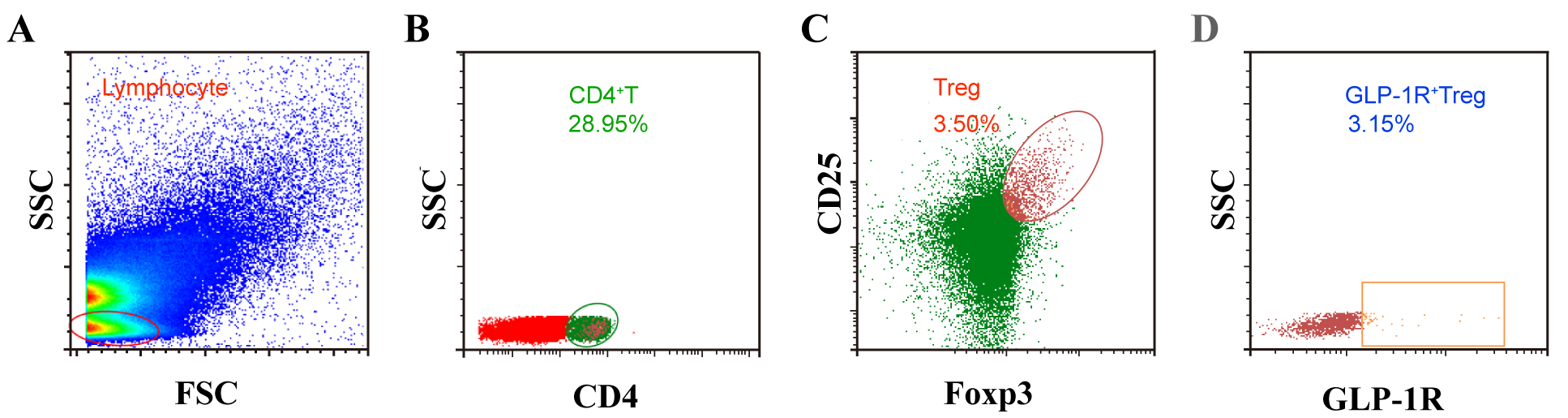
**

**Supplementary Fig. 1. Flow cytometry gating strategy for identification of Treg and GLP-1R^+^ Treg cells.**

(A)Gating on lymphocyte population based on forward and side scatter (FSC/SSC) properties ; (B) Selection of CD4^+^ T cells from the lymphocyte population; (C) Identification of CD25^+^Foxp3^+^ Treg cells within CD4^+^ T cells; (D) Detection of GLP-1R^+^ Treg cells within the Treg cell population


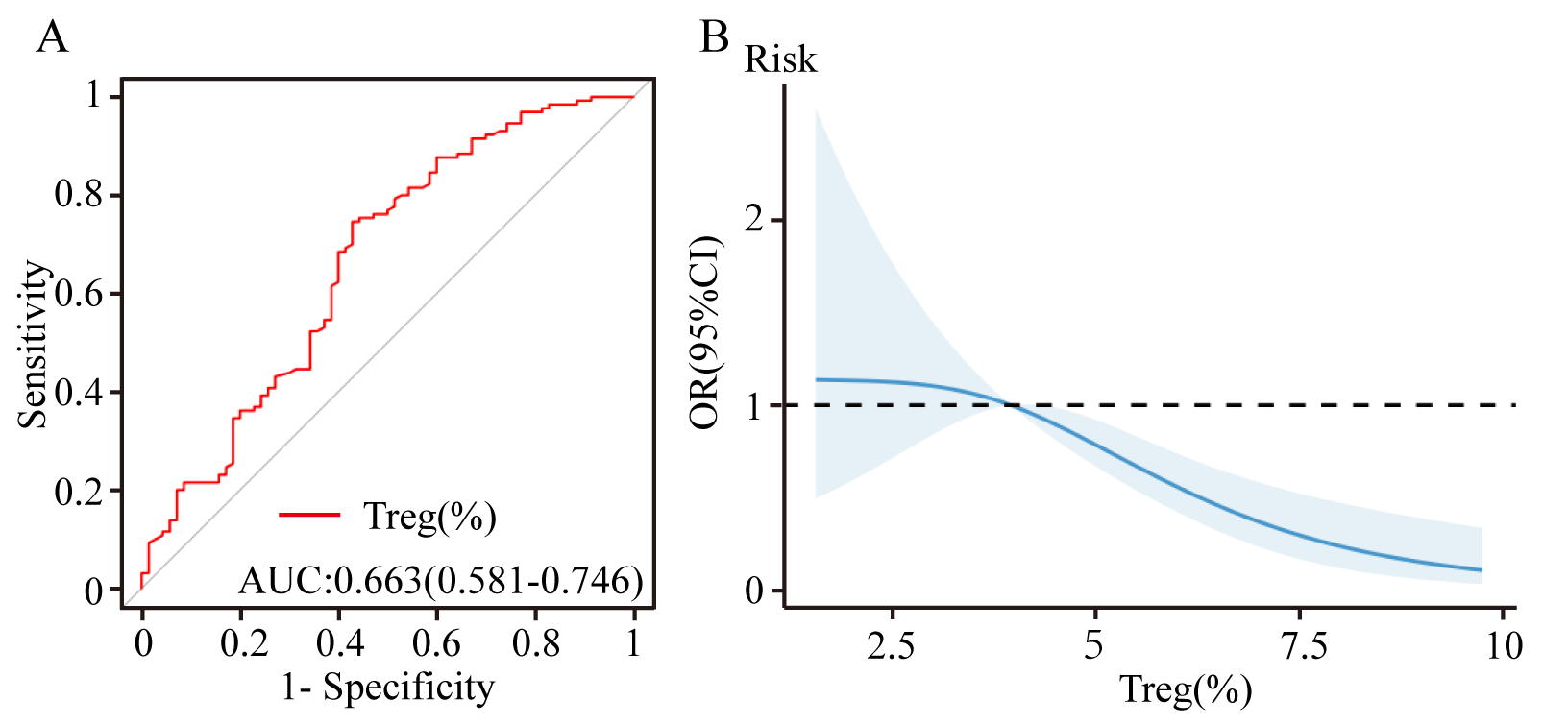


**Supplementary Fig. 2. Predictive and Dose-Response Relationships of Tregs in CHD Risk Assessment**

**(A)ROC curve analysis evaluating the discriminative capacity of Treg frequency (% of total CD4) for CHD risk stratification(AUC: 0.663) . (B) Restricted cubic spline (RCS) model illustrating the nonlinear inverse association between Treg Frequency and CHD risk.**


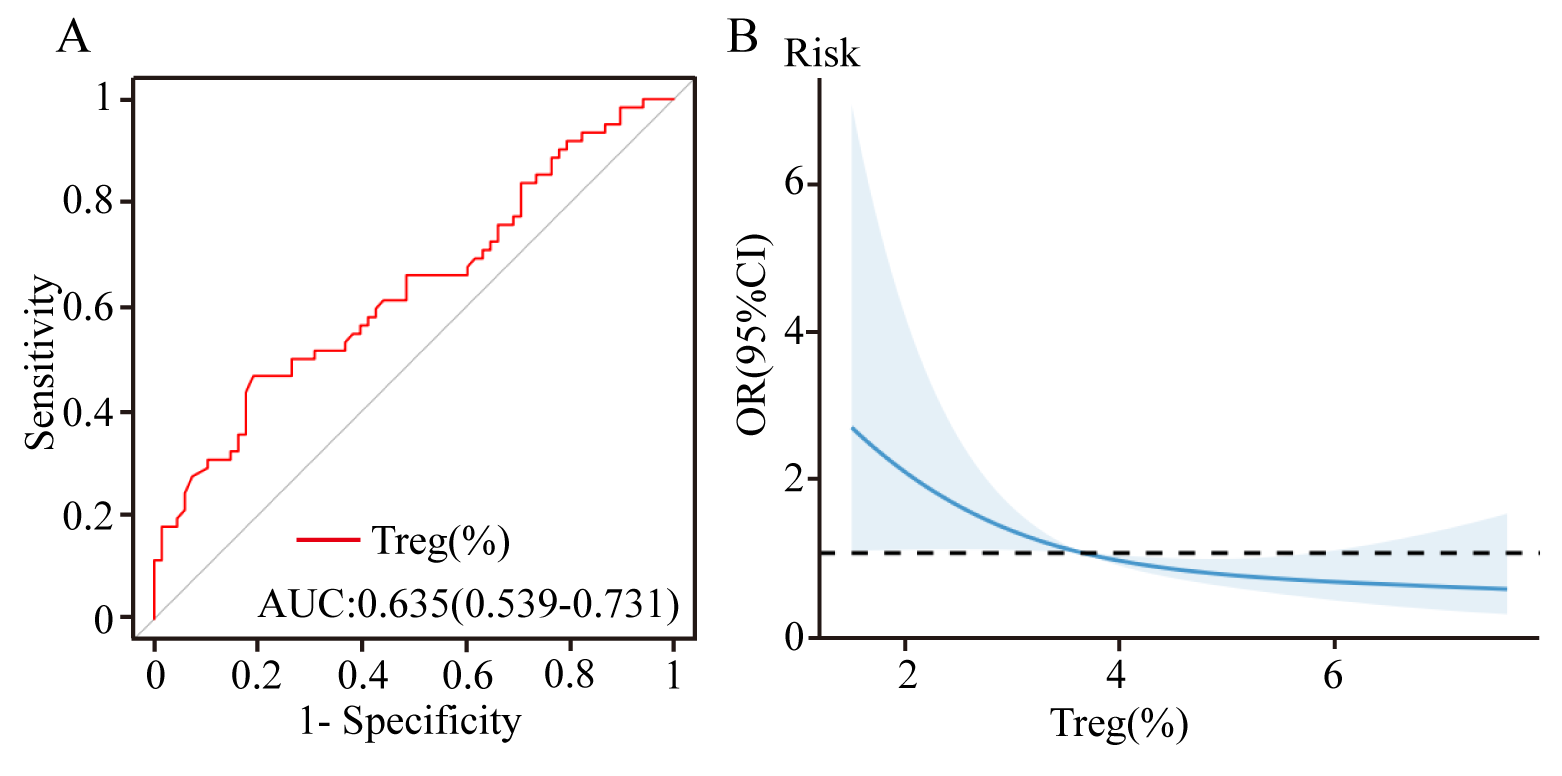


**Supplementary Fig. 3. Predictive and Dose-Response Relationship of Treg Cells proportions with Coronary Stenosis Severity**

**(A)ROC curve evaluating Treg cell proportions in predicting stenosis severity (AUC: 0.619) ;**

**(B)Restricted cubic spline plot illustrating the inverse association between Treg proportions and stenosis severity.**
